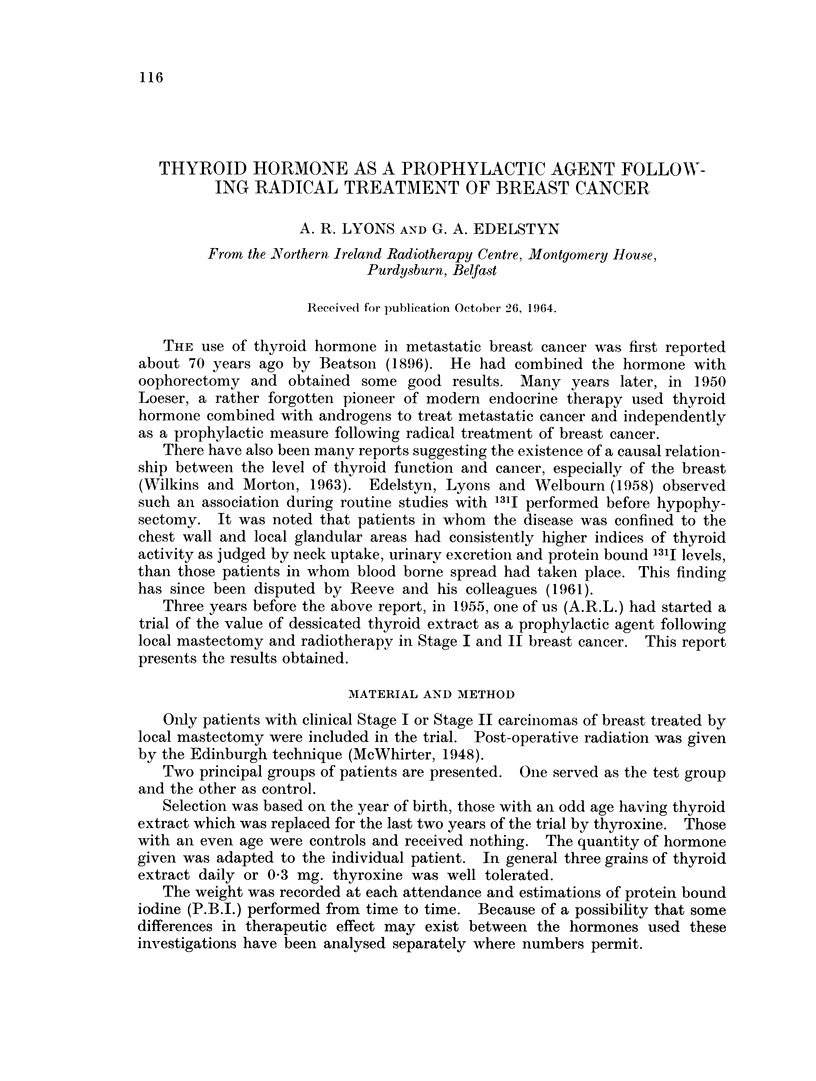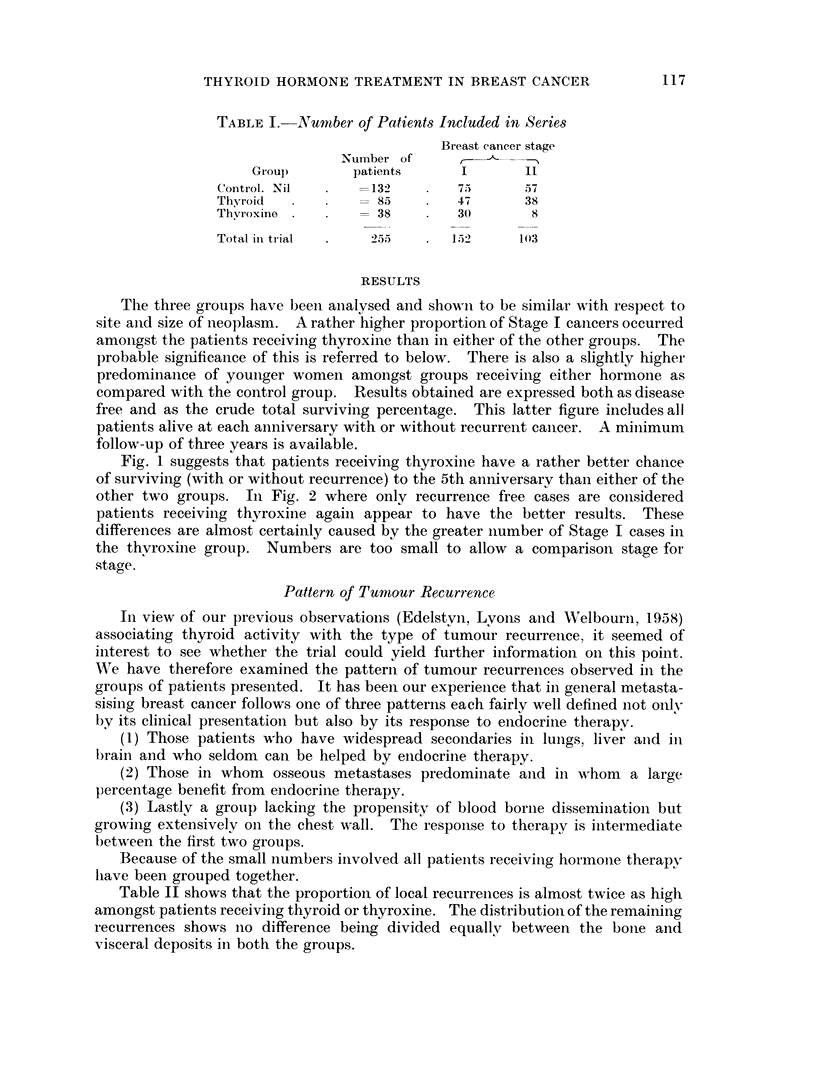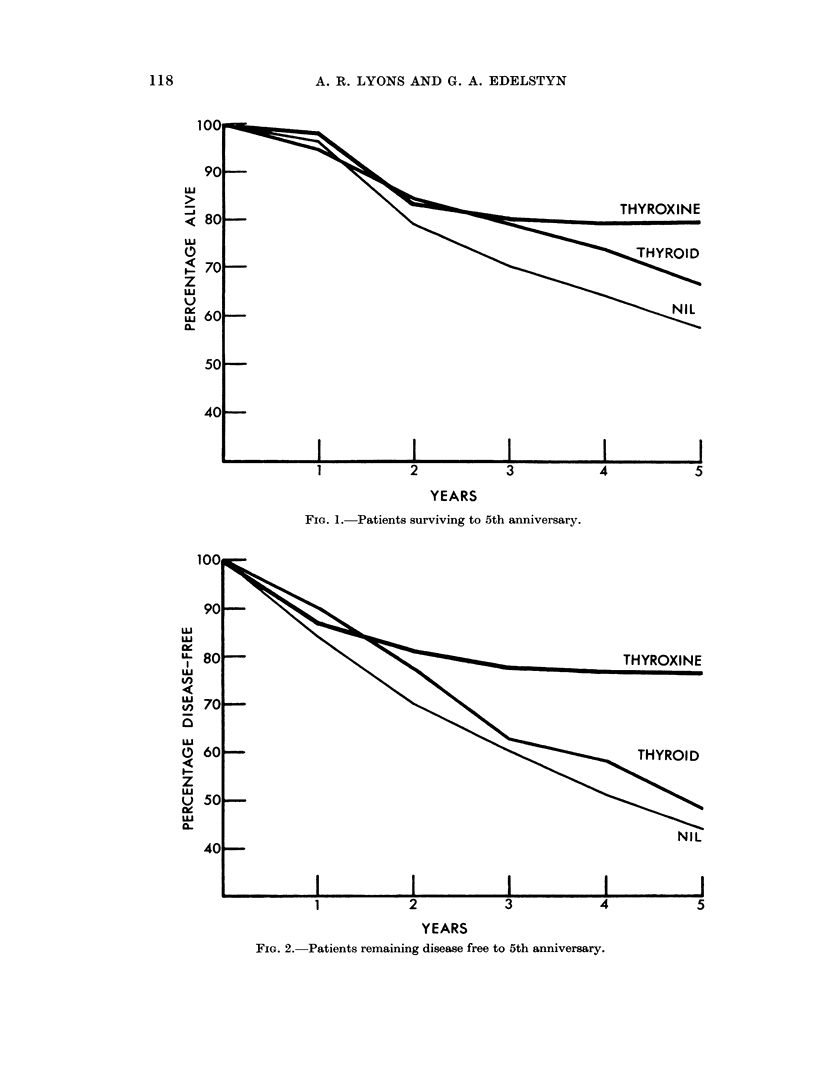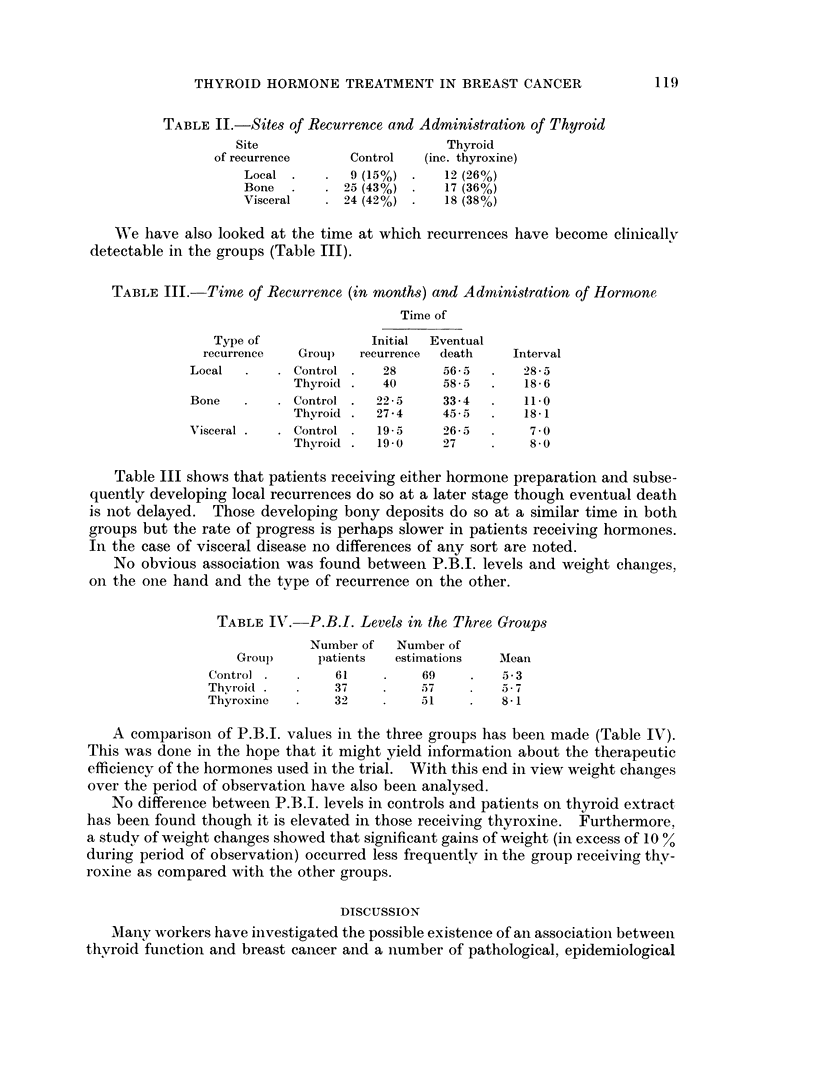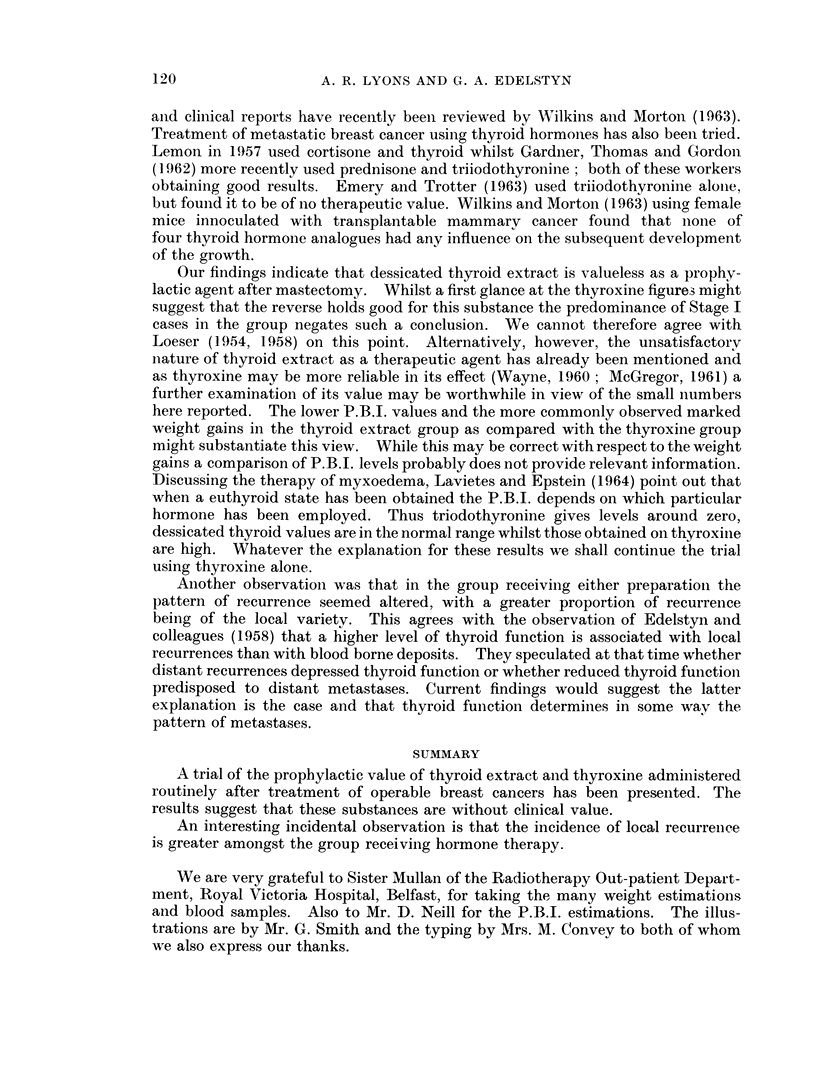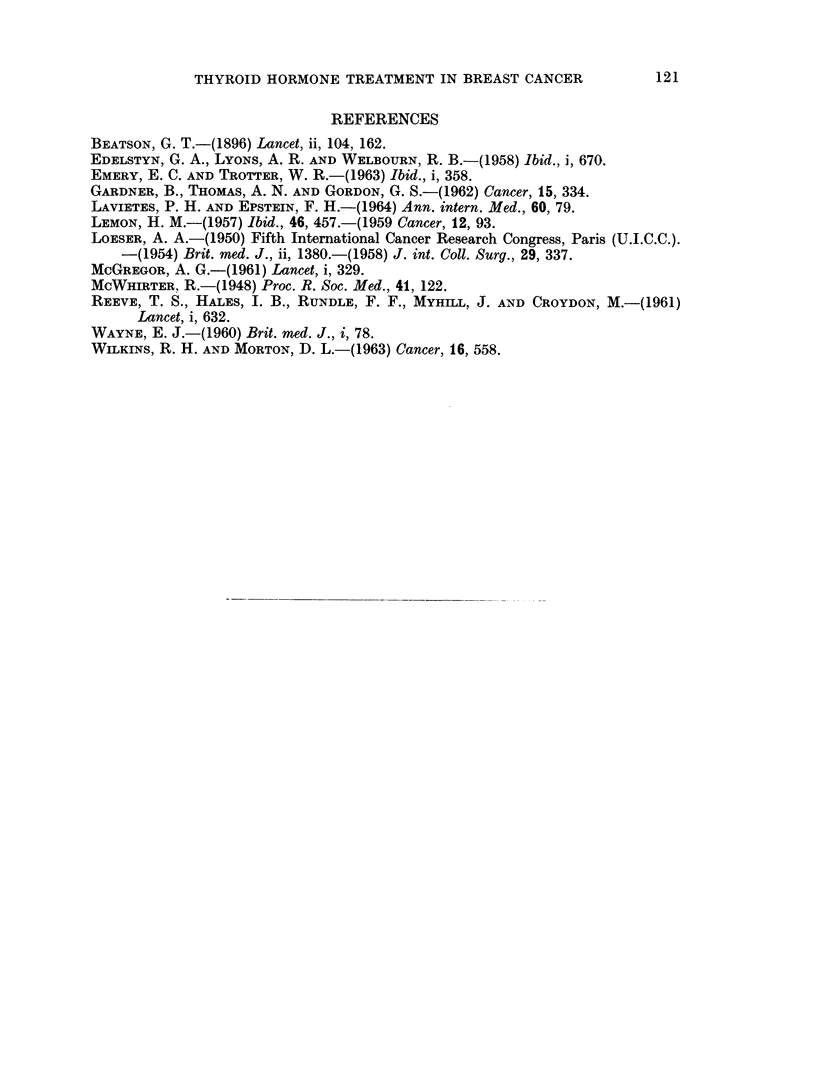# Thyroid Hormone as a Prophylactic Agent Following Radical Treatment of Breast Cancer

**DOI:** 10.1038/bjc.1965.12

**Published:** 1965-03

**Authors:** A. R. Lyons, G. A. Edelstyn


### THYROID HORMONE AS A PROPHYLACTIC AGENT FOLLOWING RADICAL TREATMENT OF BREAST CANCER

A. R. LYONS AND G. A. EDELSTYN

*From the Northern Ireland Radiotherapy Centre, Montgomery House, Purdysburn, Belfast*

Received for publication October 26, 1964.

THE use of thyroid hormone in metastatic breast cancer was first reported about 70 years ago by Beatson (1896). He had combined the hormone with oophorectomy and obtained some good results. Many years later, in 1950 Loeser, a rather forgotten pioneer of modern endocrine therapy used thyroid hormone combined with androgens to treat metastatic cancer and independently as a prophylactic measure following radical treatment of breast cancer.

There have also been many reports suggesting the existence of a causal relationship between the level of thyroid function and cancer, especially of the breast (Wilkins and Morton, 1963). Edelstyn, Lyons and Welbourn (1958) observed such an association during routine studies with $^{131}I$ performed before hypophysectomy. It was noted that patients in whom the disease was confined to the chest wall and local glandular areas had consistently higher indices of thyroid activity as judged by neck uptake, urinary excretion and protein bound $^{131}I$ levels, than those patients in whom blood borne spread had taken place. This finding has since been disputed by Reeve and his colleagues (1961).

Three years before the above report, in 1955, one of us (A.R.L.) had started a trial of the value of dessicated thyroid extract as a prophylactic agent following local mastectomy and radiotherapy in Stage I and II breast cancer. This report presents the results obtained.

### MATERIAL AND METHOD

Only patients with clinical Stage I or Stage II carcinomas of breast treated by local mastectomy were included in the trial. Post-operative radiation was given by the Edinburgh technique (McWhirter, 1948).

Two principal groups of patients are presented. One served as the test group and the other as control.

Selection was based on the year of birth, those with an odd age having thyroid extract which was replaced for the last two years of the trial by thyroxine. Those with an even age were controls and received nothing. The quantity of hormone given was adapted to the individual patient. In general three grains of thyroid extract daily or 0·3 mg. thyroxine was well tolerated.

The weight was recorded at each attendance and estimations of protein bound iodine (P.B.I.) performed from time to time. Because of a possibility that some differences in therapeutic effect may exist between the hormones used these investigations have been analysed separately where numbers permit.

TABLE I.—*Number of Patients Included in Series*

| Group | Number of patients | Breast cancer stage I | Breast cancer stage II |
|---|---|---|---|
| Control. Nil | =132 | 75 | 57 |
| Thyroid | = 85 | 47 | 38 |
| Thyroxine | = 38 | 30 | 8 |
| Total in trial | 255 | 152 | 103 |

### RESULTS

The three groups have been analysed and shown to be similar with respect to site and size of neoplasm. A rather higher proportion of Stage I cancers occurred amongst the patients receiving thyroxine than in either of the other groups. The probable significance of this is referred to below. There is also a slightly higher predominance of younger women amongst groups receiving either hormone as compared with the control group. Results obtained are expressed both as disease free and as the crude total surviving percentage. This latter figure includes all patients alive at each anniversary with or without recurrent cancer. A minimum follow-up of three years is available.

Fig. 1 suggests that patients receiving thyroxine have a rather better chance of surviving (with or without recurrence) to the 5th anniversary than either of the other two groups. In Fig. 2 where only recurrence free cases are considered patients receiving thyroxine again appear to have the better results. These differences are almost certainly caused by the greater number of Stage I cases in the thyroxine group. Numbers are too small to allow a comparison stage for stage.

### *Pattern of Tumour Recurrence*

In view of our previous observations (Edelstyn, Lyons and Welbourn, 1958) associating thyroid activity with the type of tumour recurrence, it seemed of interest to see whether the trial could yield further information on this point. We have therefore examined the pattern of tumour recurrences observed in the groups of patients presented. It has been our experience that in general metastasising breast cancer follows one of three patterns each fairly well defined not only by its clinical presentation but also by its response to endocrine therapy.

(1) Those patients who have widespread secondaries in lungs, liver and in brain and who seldom can be helped by endocrine therapy.

(2) Those in whom osseous metastases predominate and in whom a large percentage benefit from endocrine therapy.

(3) Lastly a group lacking the propensity of blood borne dissemination but growing extensively on the chest wall. The response to therapy is intermediate between the first two groups.

Because of the small numbers involved all patients receiving hormone therapy have been grouped together.

Table II shows that the proportion of local recurrences is almost twice as high amongst patients receiving thyroid or thyroxine. The distribution of the remaining recurrences shows no difference being divided equally between the bone and visceral deposits in both the groups.

FIG. 1.—Patients surviving to 5th anniversary.

FIG. 2.—Patients remaining disease free to 5th anniversary.

TABLE II.—*Sites of Recurrence and Administration of Thyroid*

| Site of recurrence | Control | Thyroid (inc. thyroxine) |
|---|---|---|
| Local | 9 (15%) | 12 (26%) |
| Bone | 25 (43%) | 17 (36%) |
| Visceral | 24 (42%) | 18 (38%) |

We have also looked at the time at which recurrences have become clinically detectable in the groups (Table III).

TABLE III.—*Time of Recurrence (in months) and Administration of Hormone*

| Type of recurrence | Group | Time of Initial recurrence | Time of Eventual death | Interval |
|---|---|---|---|---|
| Local | Control | 28 | 56·5 | 28·5 |
| | Thyroid | 40 | 58·5 | 18·6 |
| Bone | Control | 22·5 | 33·4 | 11·0 |
| | Thyroid | 27·4 | 45·5 | 18·1 |
| Visceral | Control | 19·5 | 26·5 | 7·0 |
| | Thyroid | 19·0 | 27 | 8·0 |

Table III shows that patients receiving either hormone preparation and subsequently developing local recurrences do so at a later stage though eventual death is not delayed. Those developing bony deposits do so at a similar time in both groups but the rate of progress is perhaps slower in patients receiving hormones. In the case of visceral disease no differences of any sort are noted.

No obvious association was found between P.B.I. levels and weight changes, on the one hand and the type of recurrence on the other.

TABLE IV.—*P.B.I. Levels in the Three Groups*

| Group | Number of patients | Number of estimations | Mean |
|---|---|---|---|
| Control | 61 | 69 | 5·3 |
| Thyroid | 37 | 57 | 5·7 |
| Thyroxine | 32 | 51 | 8·1 |

A comparison of P.B.I. values in the three groups has been made (Table IV). This was done in the hope that it might yield information about the therapeutic efficiency of the hormones used in the trial. With this end in view weight changes over the period of observation have also been analysed.

No difference between P.B.I. levels in controls and patients on thyroid extract has been found though it is elevated in those receiving thyroxine. Furthermore, a study of weight changes showed that significant gains of weight (in excess of 10 % during period of observation) occurred less frequently in the group receiving thyroxine as compared with the other groups.

### DISCUSSION

Many workers have investigated the possible existence of an association between thyroid function and breast cancer and a number of pathological, epidemiological and clinical reports have recently been reviewed by Wilkins and Morton (1963). Treatment of metastatic breast cancer using thyroid hormones has also been tried. Lemon in 1957 used cortisone and thyroid whilst Gardner, Thomas and Gordon (1962) more recently used prednisone and triiodothyronine ; both of these workers obtaining good results. Emery and Trotter (1963) used triiodothyronine alone, but found it to be of no therapeutic value. Wilkins and Morton (1963) using female mice innoculated with transplantable mammary cancer found that none of four thyroid hormone analogues had any influence on the subsequent development of the growth.

Our findings indicate that dessicated thyroid extract is valueless as a prophylactic agent after mastectomy. Whilst a first glance at the thyroxine figures might suggest that the reverse holds good for this substance the predominance of Stage I cases in the group negates such a conclusion. We cannot therefore agree with Loeser (1954, 1958) on this point. Alternatively, however, the unsatisfactory nature of thyroid extract as a therapeutic agent has already been mentioned and as thyroxine may be more reliable in its effect (Wayne, 1960 ; McGregor, 1961) a further examination of its value may be worthwhile in view of the small numbers here reported. The lower P.B.I. values and the more commonly observed marked weight gains in the thyroid extract group as compared with the thyroxine group might substantiate this view. While this may be correct with respect to the weight gains a comparison of P.B.I. levels probably does not provide relevant information. Discussing the therapy of myxoedema, Lavietes and Epstein (1964) point out that when a euthyroid state has been obtained the P.B.I. depends on which particular hormone has been employed. Thus triodothyronine gives levels around zero, dessicated thyroid values are in the normal range whilst those obtained on thyroxine are high. Whatever the explanation for these results we shall continue the trial using thyroxine alone.

Another observation was that in the group receiving either preparation the pattern of recurrence seemed altered, with a greater proportion of recurrence being of the local variety. This agrees with the observation of Edelstyn and colleagues (1958) that a higher level of thyroid function is associated with local recurrences than with blood borne deposits. They speculated at that time whether distant recurrences depressed thyroid function or whether reduced thyroid function predisposed to distant metastases. Current findings would suggest the latter explanation is the case and that thyroid function determines in some way the pattern of metastases.

SUMMARY

A trial of the prophylactic value of thyroid extract and thyroxine administered routinely after treatment of operable breast cancers has been presented. The results suggest that these substances are without clinical value.

An interesting incidental observation is that the incidence of local recurrence is greater amongst the group receiving hormone therapy.

We are very grateful to Sister Mullan of the Radiotherapy Out-patient Department, Royal Victoria Hospital, Belfast, for taking the many weight estimations and blood samples. Also to Mr. D. Neill for the P.B.I. estimations. The illustrations are by Mr. G. Smith and the typing by Mrs. M. Convey to both of whom we also express our thanks.

### REFERENCES

BEATSON, G. T.—(1896) *Lancet*, ii, 104, 162.
EDELSTYN, G. A., LYONS, A. R. AND WELBOURN, R. B.—(1958) *Ibid.*, i, 670.
EMERY, E. C. AND TROTTER, W. R.—(1963) *Ibid.*, i, 358.
GARDNER, B., THOMAS, A. N. AND GORDON, G. S.—(1962) *Cancer*, **15**, 334.
LAVIETES, P. H. AND EPSTEIN, F. H.—(1964) *Ann. intern. Med.*, **60**, 79.
LEMON, H. M.—(1957) *Ibid.*, **46**, 457.—(1959 *Cancer*, **12**, 93.
LOESER, A. A.—(1950) Fifth International Cancer Research Congress, Paris (U.I.C.C.).—(1954) *Brit. med. J.*, ii, 1380.—(1958) *J. int. Coll. Surg.*, **29**, 337.
McGREGOR, A. G.—(1961) *Lancet*, i, 329.
McWHIRTER, R.—(1948) *Proc. R. Soc. Med.*, **41**, 122.
REEVE, T. S., HALES, I. B., RUNDLE, F. F., MYHILL, J. AND CROYDON, M.—(1961) *Lancet*, i, 632.
WAYNE, E. J.—(1960) *Brit. med. J.*, *i*, 78.
WILKINS, R. H. AND MORTON, D. L.—(1963) *Cancer*, **16**, 558.